# Genetic analysis of 7,000 year old preserved goat leather from Cueva de los Murciélagos (Albuñol, Spain)

**DOI:** 10.1371/journal.pone.0358468

**Published:** 2026-11-04

**Authors:** Jolijn A. M. Erven, Zoé Robinet Guyet, Louis L’Hôte, Valeria Mattiangeli, Eva Fernández-Domínguez, Geordie Laidlaw, Rafael M. Martínez Sánchez, Blas Ramos Rodríguez, Maria Herrero-Otal, Pedro Henríquez Valido, José Antonio Lozano Rodríguez, The VarGoats consortium, Daniel G. Bradley, Kevin G. Daly, Francisco Martínez-Sevilla

**Affiliations:** 1 UCD School of Agriculture and Food Science, University College Dublin, Belfield, Ireland; 2 UCD Conway Institute of Biomolecular and Biomedical Research, University College Dublin, Belfield, Ireland; 3 Faculté sciences et ingénierie, Université de Toulouse, Toulouse, France; 4 Smurfit Institute of Genetics, Trinity College Dublin, Dublin, Ireland; 5 Archaeology Department, Durham University, Durham, United Kingdom; 6 Departamento de Historia, Universidad de Córdoba, Córdoba, Spain; 7 Laboratorio de Arqueología Biocultural (MEMOLab), Universidad de Granada, Granada, Spain; 8 Departamento de Historia y Filosofía, Área de Prehistoria, Universidad de Alcalá, Alcalá de Henares, Spain; 9 Centro Oceanográfico de Canarias (COC), Instituto Español de Oceanografía (IEO-CSIC), Santa Cruz de Tenerife, Spain; 10 Departamento de Historia y Filosofía, Área de Prehistoria, Universidad de Alcalá, Alcalá de Henares, Spain; 11 GenPhySE, Université de Toulouse, INRAE, ENVT, Castanet Tolosan, France; Universidad de Sevilla, SPAIN

## Abstract

Advances in ancient DNA research have expanded the range of materials from which genetic information can be recovered, enabling the analysis of atypical materials. These often preserve both host and exogenous DNA, for example due to handling or native microbes, offering a window into past diversity. Here we report one such exceptional material: a well-preserved goat leather specimen from Cueva de los Murciélagos (Albuñol, Spain), associated with an Early Neolithic context. Archaeozoological analyses show clear evidence of tanning and anthropogenic modification of the skin, while radiocarbon dating confirms it to be ~ 7,250−7,000 years old. We generated genome-wide data from the leather (Murciélagos1) and demonstrated that it derives from a domestic goat of European ancestry. Comparisons with modern goats show highest affinity to the Bermeya breed from northern Spain, demonstrating genetic connectivity in goat herds from the Neolithic to present day Iberia. We additionally recover fragmented DNA from the leather deriving from human and canid sources, the former likely reflecting handling/wearing of the leather and the latter indicating post-depositional disturbance. Murciélagos1 represents one of the oldest nuclear genome data from organic remains outside of high altitude, high latitude regions, and demonstrates the value of ancient DNA analysis in exceptional cases of organic preservation.

## Introduction

The recovery of DNA molecules from ancient and pre-modern biological remains has transformed our understanding of the past [1–4]. Such ancient DNA (aDNA) studies are of an ever-increasing scale but typically focus on specific material types. These sources are well justified by the taphonomic realities of what tends to be preserved (i.e., inorganic tissue) and in which DNA best survives. In exceptional cases, preservation conditions allow other materials, such as organic tissues, to remain relatively intact for hundreds or even thousands of years. Indeed, the first reported ancient DNA sequence was from a relatively recent historical dried quagga († *Equus quagga quagga* Boddaert) muscle tissue (collected ~1844 CE [5]). Environments, such as permafrost and anhydrous locations, can postpone the typical post-mortem degradation of soft tissues. There are several instances of thousands-of-years-old hair providing endogenous DNA molecules [6–10]. Preserved skin has also proven a rich source of ancient biological inference, including genetic data from historic dogs [11,12], rock pigeons (*Columba livia* Gmelin) [13], and Egyptian mummies [14,15]; gene expression patterns of the Tasmanian tiger († *Thylacinus cynocephalus* (Harris)) [16] and the woolly mammoth († *Mammuthus primigenius* (Blumenbach)) [17]; and the genomic architecture of the woolly mammoth [8]. Permafrost conditions have also allowed the recovery of aDNA from soft tissue of a Late Pleistocene wolf [18]. At lower latitudes, a Neolithic legging of goat skin from the Swiss alps and a naturally-mummified sheep leg from the salt mine of Chehrābād, Iran, provided a 4,200 and 1,600 year old genome, respectively [19,20]. While warm climates generally reduce DNA preservation in organic tissues, recovery is occasionally still possible. Ancient DNA has been successfully extracted from mummified human hair of a ~ 4,000-year-old individual from Sudan [7] and from a hair fragment and cloak in China of similar age, identifying them as goats [21]. Nevertheless, the recovery of aDNA from organic tissues in warmer regions remains rare and might be limited to well-preserved specimens or cave specimens.

One case of exceptional preservation is the Cueva de los Murciélagos site (Albuñol, Granada) in southern Iberia (Fig 1A), where there is outstanding preservation of organic materials ascribed to different phases of Late Prehistory by desiccation (Fig 2 [22,23]). The site is a karstic cave in the lower part of the Angosturas Gorge at an altitude of 450 m and 7 km from the Mediterranean coast. The shelter of the cave is 15 m wide with an east-facing entrance; the interior part is around 60 m long and 30 m wide, with a 48 m drop. The first information about Cueva de los Murciélagos dates from 1857, following the interest shown by a mining company in extracting galena from inside the cave. These workers recovered partially mummified human remains, as well as clothing, baskets, and other wooden objects. In addition, a large number of plant artefacts, lithic tools, and ceramics were retrieved, as well as archaeozoological remains [24]. Unfortunately, these objects were found disturbed in the cave at the time of their discovery; the majority are now deposited at the Museo Arqueológico Nacional de Madrid (MAN) and the Museo Arqueológico y Etnológico de Granada (MAEGr). Radiocarbon dating have placed the sequence of the cave funerary use, with a first phase between 9,500−9,000 cal BP (Mesolithic – hunter-gatherer populations); a second phase between 7,200−6,700 cal BP (Early Neolithic – first farming populations); a third phase between 6,700−6,000 cal BP (Middle Neolithic); and a fourth phase between 6,000−5,700 cal BP (Late Neolithic). Finally, there is a Bronze Age period between 3,900−3,700 cal BP. The Cueva de los Murciélagos site has been interpreted as a burial place in those Late Prehistoric periods, where goods placed likely have a symbolic significance [22,23].

**Fig 1:**
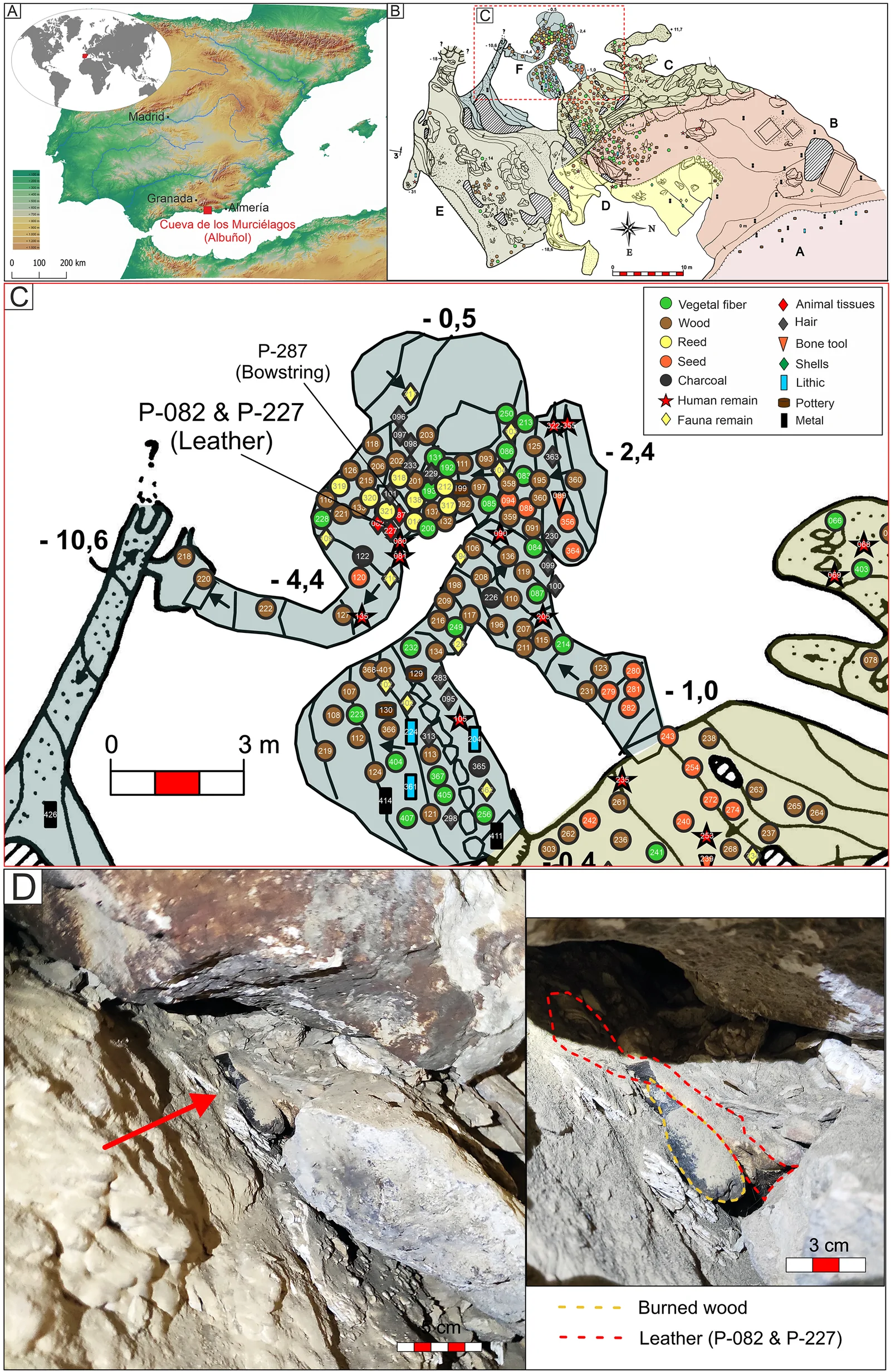
Cueva de los Murciélagos of Albuñol. A) Location of Cueva de los Murciélagos (Albuñol) in the south of the Iberian peninsula; B) Cave plan showing the delimitations used for the survey and for mapping the distribution of archaeological materials (modified from the free access original topography made in 1984 by Gónzalez Rios, M., Menjibar Silva, J.L. and García Ligero, M.; C) Close-up of Sector F showing the location of the tanned skin/leather studied in this paper and the bowstring, both of which were dated; D) Photograph of the leather at the moment of its discovery, embedded in a stone wall built by 19th-century miners (outlined in red).

**Fig 2:**
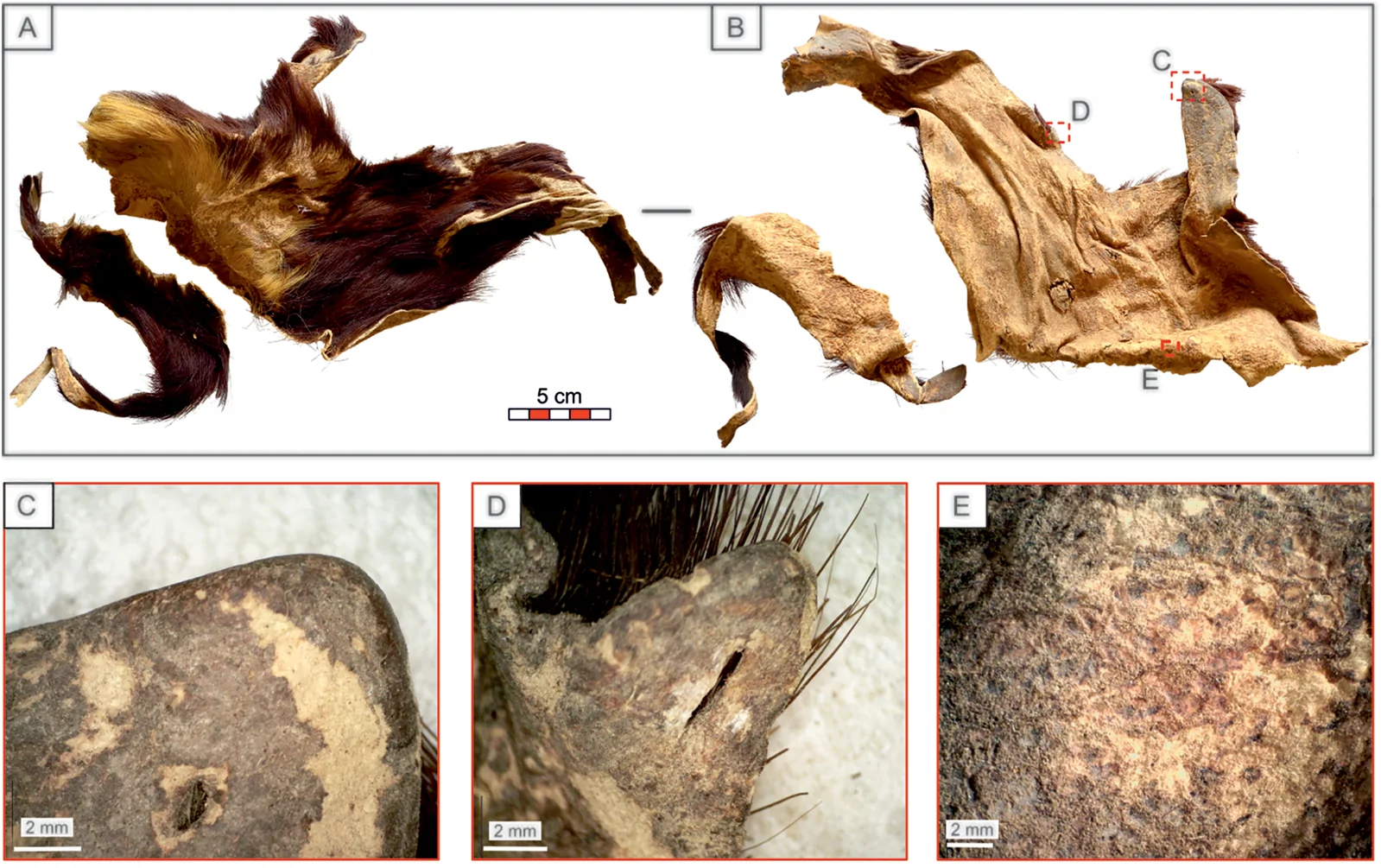
Goat leather from Cueva de los Murciélagos of Albuñol. A) hair side of leather; B) flesh side of leather; C & D) Example of anthropic perforation and tanned edges; E) Tanned surface with traces of softening from use.

The fieldwork in the cave, in the framework of the MUTERMUR project, was carried out as an intensive survey, and allowed for the recovery of different archaeological materials in the miners’ waste dumps (Fig 1B). During the surface survey, two fragments of animal skin (CM22/SF/P-082 and CM22/SF/P-227) were recovered. Initial archaeozoological assessment of the skin ascribed it to the genus *Capra*. The animal skin is attributed to the Early Neolithic phase (7,200−6,700 cal BP), which represent one of the earliest farming communities within the region. A recent study on aDNA from Early Neolithic Iberian sheep has shown continuity with Anatolian lineages [25]; nothing is currently known about Neolithic Iberian goats. Ancient DNA from these early herds has the potential to reveal the origins and movements of these pioneering communities.

Accordingly, the objective of this study is to evaluate the biological and chronological identity of this exceptional skin object from Cueva de los Murciélagos, named Murciélagos1, to determine whether it is a Neolithic representative of the domesticated species *Capra hircus* L. or the wild species *Capra pyrenaica* Schinz. To answer this question, we combine population genetic analyses, aimed at providing biomolecular evidence for the specimen’s taxonomic classification, with radiocarbon dating, to situate the specimen within its archaeological and temporal context. Through this integrated approach, we aim to shed light on the exploitation and management of goats during the Neolithic in Iberia.

## Results

The materials analyzed here correspond to two different fragments of the same animal skin (Table S1 in S2 File). Fragment P-082 is an irregularly and asymmetrically tanned hide – from this point referred to as leather – tending towards a rectangle measuring 346 mm long by 202 mm wide. The second fragment P-227 is a piece of 226 mm long by 138 mm. The two leather fragments were found together beneath a block displaced by miners in a deep area of the cave, defined as Sector F (Fig 1C and 1D). The fragments likely belong to the same individual, based on their direct spatial association, similar thickness and texture, comparable hair follicle pattern, and shared taphonomic features, all of which suggest a common origin and depositional history. Species identification was based on the morphology and distribution of hair follicles observed on the grain surface of the leather. Goat skin is characterized by a single row of primary hair follicles associated with one or two secondary follicles, a pattern that distinguishes it from cattle and deer [26]. Although it has not been possible to physically refit the break, both fragments exhibit fresh fracture surfaces along the areas that were likely originally joined. The leather retains hair on one surface, while the opposite side displays traces consistent with tanning treatment as well as four perforations showing clear evidence of anthropogenic modification (Fig 2). The flesh side exhibits a smooth and compact surface with the absence of adhering soft tissues and a homogeneous appearance of the collagen matrix. These characteristics are consistent with processed leather (Fig 2E). The perforations have been identified as anthropogenic, based on the distinction that breaks produced by insect activity exhibit irregular, stepped edges typical of gradual biological degradation [27,28], while anthropogenic perforations show smooth, sharply defined margins consistent with deliberate human modification (Fig 2C and 2D).

The radiocarbon dating of the Cueva de los Murciélagos leather (Ua-78252) – from this point referred to as Murciélagos1 – yielded an age of 7,246−7,008 cal BP (2σ calibrated range; Fig 3C). In the context of the cave’s chronological framework, this estimated age places this object within the earliest phase of the Early Neolithic (Fig S1 in S1 File and Table S2 in S2 File). This phase of material deposition has been identified in other objects, such as bowstrings (7,239−6,999 cal BP [23]). Together with the presence of human remains, this suggests that the area where the leather was found was used as a burial site during the Early Neolithic, and that these objects were deposited as grave goods.

**Fig 3:**
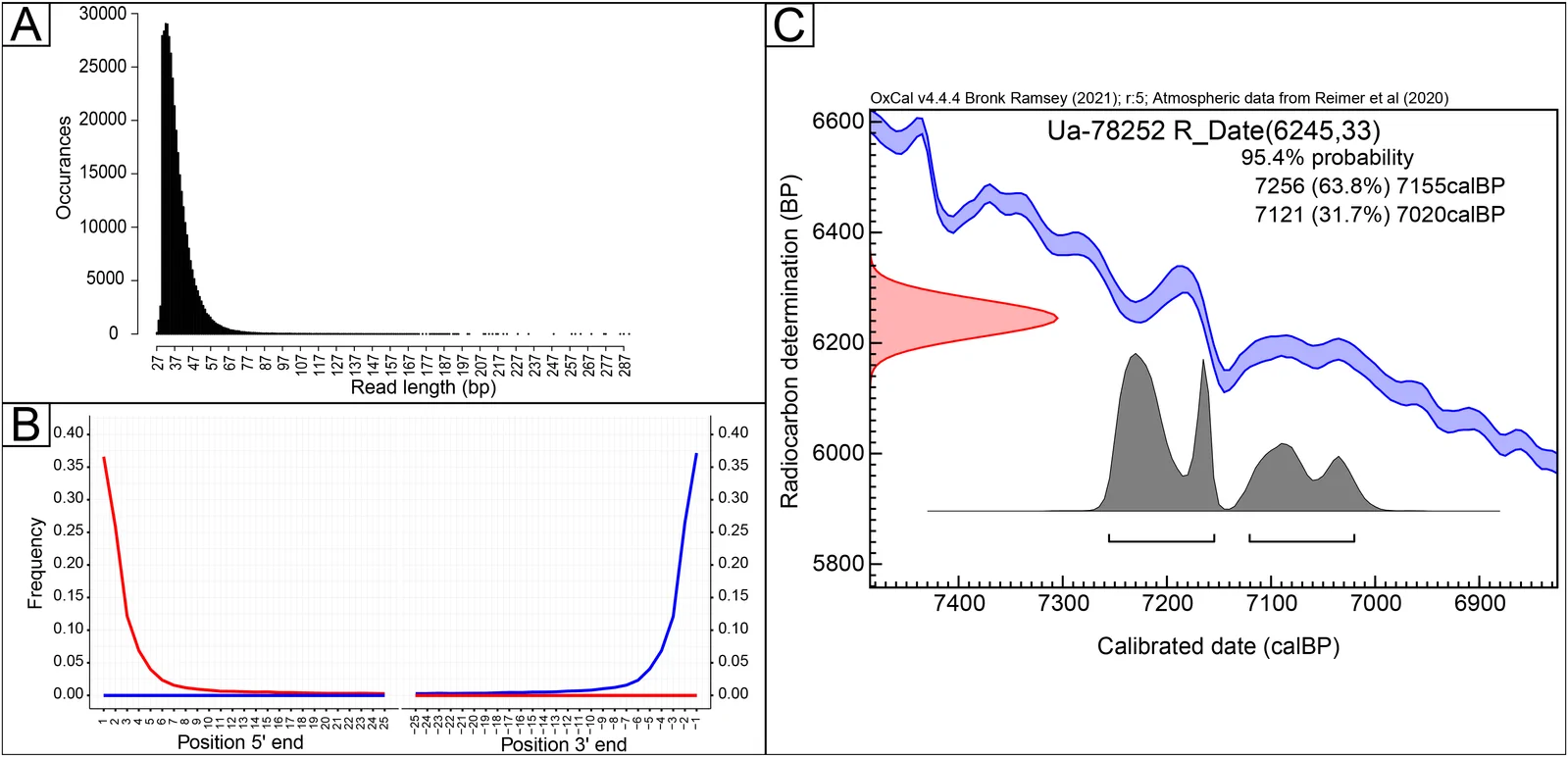
Temporal assessment of Murciélagos1. A) Read length distribution; B) aDNA damage, C > T (red) and G > A (blue) frequency of misincorporation rates at the 3’ and 5’ end of reads; C) Radiocarbon date calibrated in OxCal 4.4 software [29] using the IntCal20 terrestrial calibration curve [30].

The leather exhibits clear signs of anthropogenic modification, suggesting deliberate processing and shaping. In a funerary context, such a treated hide may have functioned as a wrapping or covering for the deceased, or as an intentionally deposited element of the funerary assemblage. Alternatively, it may represent a garment or item of clothing associated with the body at the time of burial. While its precise function cannot be determined, the contextual and technological evidence supports its intentional incorporation into mortuary practices.

To determine if Murciélagos1 was domestic *Capra hircus*, wild *Capra pyrenaica* or another species of Bovidae, we aligned sequencing reads across a set of reference genomes using FastQ Screen [31] (Table S3 in S2 File). Initial assessment indicated that most reads were assigned to the goat ARS1 genome (433,227 reads, 46% of total mapped reads, Table S1 in S2 File). However, we also detected some reads aligning to either human and fox reference genomes (Tables S1 and S4 in S2 File). To account for this we removed reads aligned to the goat genome which also aligned to either the fox or human genomes (161,320 and 344,827 reads, contributing 17% and 37% of total mapped reads respectively, Figs S2–S6 in S1 File), leaving 377,825 goat-aligned, unique reads (40% of total mapped reads) after other quality control steps. In sum, we retrieved both mitochondrial DNA (36.68✕) and autosomal DNA (0.0049✕) from the preserved leather. Aligned reads showed a short average length (37 bp) and substantial postmortem damage patterns compatible with aDNA (Figs 3 and S3–S7 in S1 File). We determined the molecular sex of Murciélagos1 to be female (Supplementary Note 1 and Figs S7 and S8 in S1 File).

We additionally characterized the metagenomic content (see Supplementary Methods in S1 File) of the non-mammalian reads: the majority fall within the Eukaryota domain (90.4%), and more specifically within the kingdom Fungi (88.1%), with a predominance of the phylum Ascomycota (82.1%; Fig S9 in S1 File), which is one of the most dominant Fungi in caves [32]. We additionally assessed human-assigned reads: after merging all human-aligned reads and genotyping with nf-core/EAGER ([33], Supplementary Note 2 in S1 File and Table S4 in S2 File), 7,261 SNPs overlapped with the 1,352K SNPs in the Ancient Human DNA Target Enrichment panel (Twist Bioscience), while 133 reads aligned against human mtDNA (Table S4 in S2 File). Molecular sex of the human DNA was assigned as male; however, due to the low number of reads this assignment should be considered tentative (Fig S10 in S1 File). Aligned reads showed a short average length (68 bp) and substantial postmortem damage patterns compatible with aDNA (Figs S4 and S5 in S1 File). Due to insufficient coverage of potentially informative SNPs and lack of phylogenetic resolution, no further analyses were conducted with the human reads.

To probe if the genetic ancestry of Murciélagos1 was consistent with that of a Neolithic Iberian domestic goat, we computed a projection PCA using LASER 2.0 [34]. Using the VarGoats modern genome dataset filtered for a MAF 5% as a background reference (Table S5 in S2 File, 2,420,382 SNPs [35],), Murciélagos1 (9,127 SNPs) falls within European ancestry (Fig 4), close to modern southern European breeds (Swiss, Italian and French), and overlapping with the modern Swiss breed cluster. In contrast, modern day Spanish breeds are shifted away from the European cluster towards African breeds. From these, the available northern Spanish breed (Bermeya) is closer to the European cluster, while the Palmera breed from the Canary Islands falls within the African cluster (Fig S11 in S1 File). This genetic affinity between the Spanish goat from the Balearic islands and Catalonia with African goat populations, are thought to have originated through introgression from African goats into Spanish breeds [36,37]. To further test the domestic status of Murciélagos1, a *D* statistic test (*D*(Out, Neolithic Goat; *Capra pyrenaica*, Modern breeds)) was performed, showing that Murciélagos1 has a closer genetic affinity to modern domestic breeds than with wild *Capra pyrenaica* (Iberian ibex; Fig S12 in S1 File and Table S6 in S2 File; *D* statistic range 0.068-0.080, Z values 34–234), consistent with Murciélagos1 representing a domestic goat.

**Fig 4:**
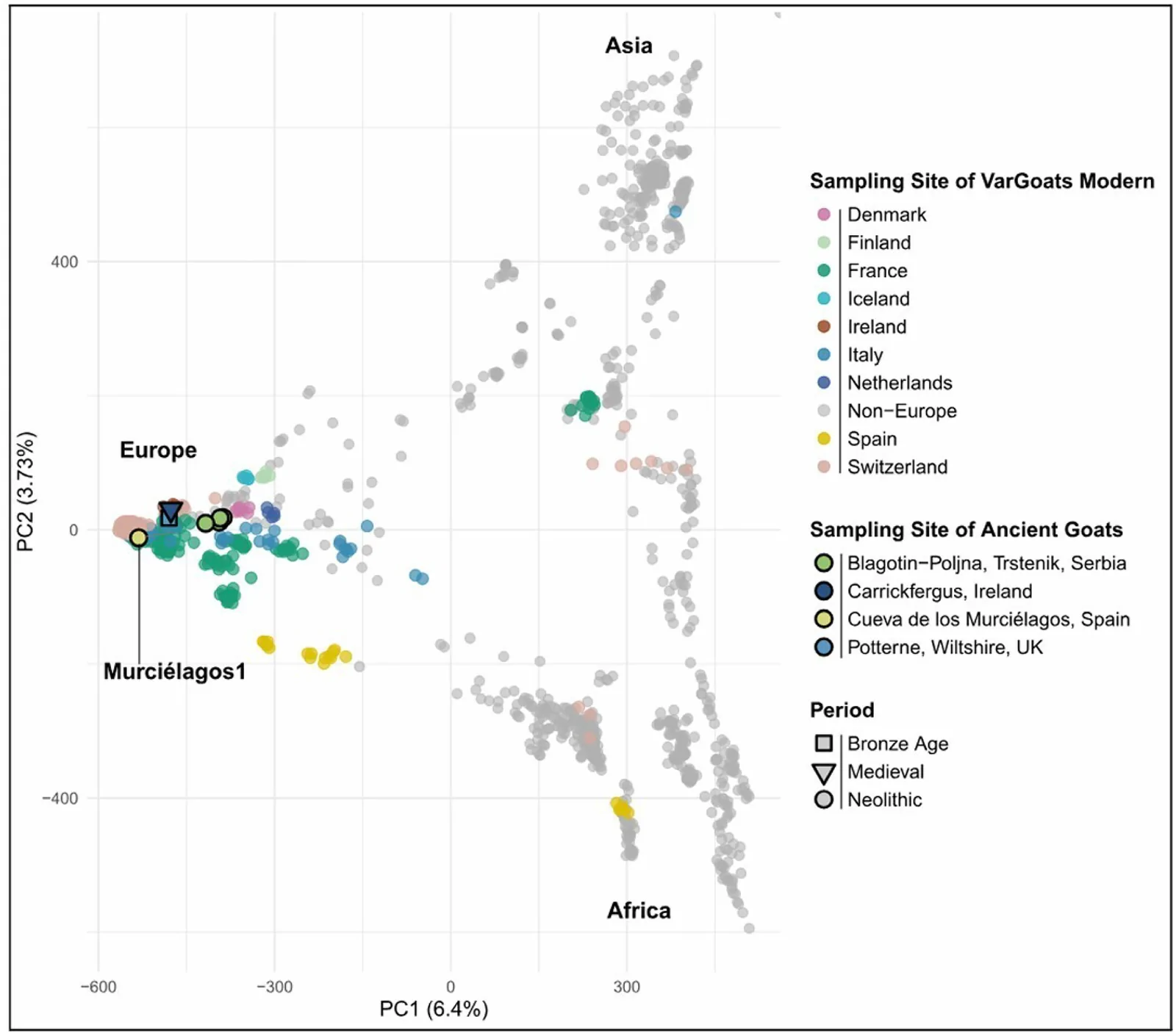
Principal component analysis of the VarGoats Modern dataset and ancient European goats, including our study sample Murciélagos1 goat leather. Each point represents the genetic information of a goat sample. Axes PC1 (6.4%) and PC2 (3.73%) represent the first two principal components, which explain the genetic variation. Grey points correspond to modern non-European samples; those located at the bottom right of the PCA form the African cluster, and those at the top right form the Asian cluster.

Given the low total number of aligned reads, we sought to further confirm the relationship between Murciélagos1 and modern goat breeds. To do this we performed an outgroup *f*_*3*_ analysis to measure the amount of shared genetic drift between Murciélagos1 and modern goat breeds. Consistent with PCA results, Murciélagos1 showed more genetic affinity with European breeds compared to African and Asian breeds (Fig 5). Notably, the Bermeya breed (BEY) from northern Spain show the highest shared genetic drift with Murciélagos1 (Table S7 in S2 File). The Valais Copper Neck (Swiss) and Poitevine (French) breeds also had high genetic affinity with Murciélagos1. In summary, these results suggest a degree of population continuity between the Murciélagos goat and both Spanish and European goat breeds today.

**Fig 5:**
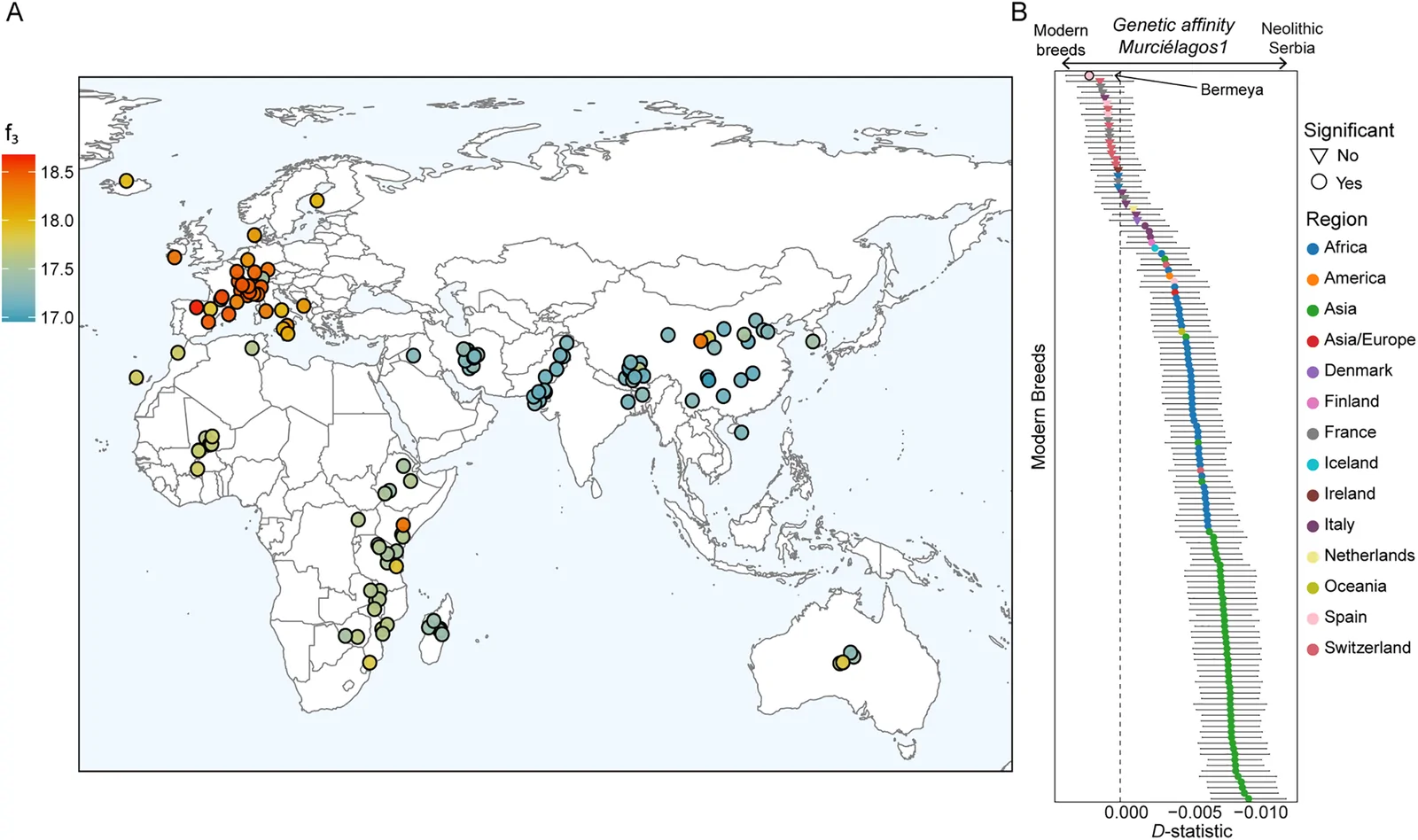
Outgroup *f*_*3*_ and *D* statistics for Murciélagos1. A) Geographical distribution of shared genetic drift (outgroup *f*_*3*_) between the Neolithic Murciélagos1 goat from Iberia, and modern domestic goat breeds. The range of the outgroup *f*_*3*_ statistic in modern domestic comparisons ranges from 15 to 30. B) *D* statistics of the form *D*(Out, Murciélagos1: Neolithic Serbia (Blagotin), Modern breeds). This *D* test measures affinity of Murciélagos1 with Neolithic Serbia or modern breeds. Negative indicates closer affinity (shared derived alleles) of Murciélagos1 to Blagotin and positive indicates closer affinity towards the modern breed in question.

We then used *D* statistics to confirm the close genetic affinity between Murciélagos1 and the northern Spanish Bermeya breed. We tested if Neolithic goats shared more genetic affinity with the Bermeya breed compared to other modern breeds (*D*(Sheep, Neolithic goat populations; Bermeya, Modern breeds), using Murciélagos1, Serbian genomes (Blagotin-Poljna, ~ 7936−8190 BP [38]), and an East Iranian genome (Tepe Sang-e Chakhmaq, ~ 8,055 BP [38]) as comparisons. Murciélagos1 showed greater genetic similarity to the Spanish Bermeya breed than to other breeds today (Fig S13 in S1 File and Table S8 in S2 File); *D* statistics ranged from −0.00081 to −0.012 (Z value ranged from −1.14 to −18, 5 breeds did not reach significance at |Z| > 3). However, this trend was not significant for some southern European breeds, which also displayed strong similarity with Murciélagos1 in the outgroup *f*_*3*_ analysis. In contrast, a Neolithic Serbian goat showed greater genetic similarity to southern and central European breeds rather than to Bermeya goats, while a Neolithic Iranian genome displays low genetic affinity to modern European breeds. This illustrates that Neolithic goats had varying degrees of relatedness to current-day European breeds and indicate population continuity across Europe and validating the genetic connection between Murciélagos1 and Spanish goats today.

We additionally tested the relative sharing of derived alleles between Murciélagos1 and either the closest available Neolithic goats (Blagotin-Poljna, Serbia) versus the modern breeds with the *D* statistics test *D*(Out, Murciélagos1; Neolithic Serbia, Modern breeds), then ranked results based on degree of greater allele sharing with Murciélagos1. The Bermeya breed showed highest relative allele sharing with Murciélagos1 among modern breed comparisons (Fig 5B and Table S9 in S2 File, *D* = 0.0023, Z = 4). Other southern European breeds had a similar pattern of relatively high allele sharing with Murciélagos1, although their test statistics did not reach significance likely due to the low sequencing depth available for Murciélagos1 (*D* statistic range 0.0015-0.000654, Z score 2.52-1.12). Combined, these analyses demonstrated that Murciélagos1, unlike other published ancient goats, had a high genetic affinity with Spanish breeds today, particularly the Northern Spanish Bermeya breed. This suggests that Murciélagos1 belonged to or was related to a population that gave rise to or with a direct genetic connection to Spanish goat breeds today. A final *D* statistic of the form *D*(Out, Neolithic; Modern Moroccan, European/African modern breeds) demonstrated that Murciélagos1 shows no genetic affinity towards African breeds (Fig S14 in S1 File and Table S10 in S2 File).

Finally, we assessed the mitochondrial genome of Murciélagos1, placing it within the known diversity of modern and other ancient goat samples. Murciélagos1 belongs to haplogroup A (Fig S15 in S1 File), the haplogroup most commonly observed in European goats in the Neolithic and today [38]. The bootstrap value at the haplogroup A node was 95, which provides strong support for this assignment. However, the precise position of Murciélagos1 within this haplogroup was not well resolved, with low bootstrap values, indicating uncertainty regarding its relationship to other sequences within haplogroup A.

## Discussion

Murciélagos1 (~7,250−7,000 years old) represents the to-date oldest nuclear genomic data reported from dessicated preserved skin remains outside of permafrost or frozen context. The leather clothing of the Tyrolean Iceman (~5,300 BP) has yielded mtDNA, but these derive from a glacial context [39]. Similarly, mtDNA amplification has been reported of Neolithic leggings made of goat skin, dating to ~4,700 BP, but were found in high altitude context [20]. DNA has also been recovered from the dessicated soft and hard tissues of Egyptian mummies, but these are comparatively recent (~3,400−1,500 BP) [15], as are reports of genetic data from a variety of tissues from Egyptian ibis (*Threskiornis aethiopicus* (Latham)*)* [40*]*. Additionally, 4,000 year old DNA has been reported from preserved human hair, from Greenland [41] and Sudan [7]. Finally, genomic data has been obtained from a naturally mummified sheep leg, but dates to ~1,600 BP [19]. In contrast, Murciélagos1 (~7,200 years BP) represents skin tissue preserved through desiccation in a low latitude, cave environment, which has retained sufficient genetic data for nuclear genome exploration.

As expected for a specimen of this age and preservation context, DNA preservation was poor, resulting in short read lengths and extensive fragmentation. Despite these inherent challenges, we recovered sufficient endogenous DNA to generate a genome that was of adequate quality for population genomic analyses. We further minimized the potential effects of DNA degradation by applying stringent quality-control filters, damage-aware analytical approaches, and validated results by downsampling experiments, increasing confidence in the robustness of our results. Nevertheless, the fragmented nature of the genome inevitably limits the resolution of certain analyses, particularly those aimed at detecting subtle ancestry components or weaker admixture signals. Consequently, while the patterns observed here provide important insights into the genetic affinities of Neolithic Iberian goats, they should be viewed as an initial framework that will benefit from validation as additional high-quality ancient genomes become available.

This discovery highlights the possibility of effective recovery of aDNA from low-latitude cave material, where site-specific microenvironmental conditions enabled unexpected organic preservation. Our results underscore the value of broadening substrate selection beyond traditional elements such as the petrous, where such materials are available. By targeting alternative tissues, we not only recovered host aDNA but also obtained a broader metagenomic profile, allowing for new questions to be asked of life in the past. Exceptionally preserved specimens may further expand our opportunities for investigating tissue-specific epigenetic patterns [42] and potentially even RNA research [16,17]. Future research should be equally cognizant to minimize destructive sampling, for example by systematically testing minimally invasive approaches such as surface rubbings, which have been successfully applied in parchment aDNA research [43,44].

While study of the materials is on-going, an initial assessment of the faunal bone assemblage from Cueva de los Murciélagos sheds light on Neolithic strategies for selecting and processing animal raw materials. There was marked preference for long and flat bones from medium and large ruminants – particularly domestic goats, but also bovids and red deer [45]. Goat metapodia and tibiae were frequently chosen to craft pointed implements, spatulas, and toothed tools, using techniques such as percussion, splitting, abrasion, and longitudinal grooving. Traces of intensive use and microscopic wear patterns underscore both the functional efficiency of goat bones and the advanced craftsmanship of the prehistoric makers [46]. Additionally, bowstrings composed of animal sinews and preserved at Cueva de los Murciélagos were identified by ZooMS as *Capra* species [23]. Radiocarbon dating of a cord (Beta-684048) showed a date between 7,239−6,999 cal BP, contemporary with Murciélagos1. Our genetic confirmation that Murciélagos1 is a domestic goat provides evidence that, at least for this specimen, domestic flocks were exploited in the development of this material culture, rather than Iberian ibex (*Capra pyrenaica*) indigenous to the Peninsula prior to the arrival of husbandry.

The introduction of managed animals to Iberia marked a pivotal shift in subsistence strategies, reflecting the spread of Neolithic husbandry and human-animal interactions across the region. Within a few centuries, livestock herds came to constitute the majority percentage of zooarchaeological assemblages in the Mediterranean and southern Iberia during the Early Neolithic [47–50]. It does not seem possible to draw reliable patterns by considering the Peninsula as a whole [51]. In fact, livestock herding does not seem to play a major role in assemblages from the northern and northwestern Peninsula until the following millennium (7th millenium BP), when the importance of domestic herds approaches that observed in other Iberian regions [49,52]. From the Early Neolithic onwards, coastal sites continue to show faunal spectra inherited from hunter-gatherer economies, to which the domestic component was gradually incorporated, as seen at Cova de les Cendres (Alacant, eastern Iberia) [53], Nerja (Vestíbulo and Torca sites) [54], and Zacatín (Gualchos-Castell de Ferro) [55] in the southern Iberian Peninsula.

The genetic data recovered from Murciélagos1 offers the first glimpse at the genetic makeup of goat populations from Neolithic southern Iberia. This piece shows genetic ancestry consistent with European domestic goat populations, in agreement with the archaeological evidence of the use of managed animals and crops at the site [22,56]. Murciélagos1 has the highest affinity with modern southern European breeds, particularly with the northern Spanish Bermeya breed. This implies that Murciélagos1 was part of, or closely related to, a population that was at least partially ancestral to the Bermeya breed. Yet, Murciélagos1 does not show a high affinity with southern Spanish breeds, likely a consequence of later African introgression in these breeds [36,37]. While there is African ancestry in Bermeya goats, it is notably lower compared to the other southern Spanish breeds [36,37]. A limitation of this study is the low representation of modern Spanish breeds, with only four breeds (two from the Iberian Peninsula, one from the Balearic Islands, and one from the Canary Islands). Future analyses with access to a broader range of native Iberian breeds may improve our understanding of the genetic affinities between Neolithic goats and modern Iberian breeds, and possibly identify regional patterns of continuity that are not captured by the current dataset.

The presence of African ancestry in Spanish goat breeds is evident, yet the question on when this happened remains unclear. There is archaeological and paleogenomic evidence of connections between the southern Iberian Peninsula and the northwest coast of Africa [57–60], and one study indicates a synchronous emergence of the Neolithic in southern Iberia and northwestern Africa [58]. Earliest evidence of domestic goats in southern Iberia is dated to ~7,500 B P [61], ~ 7,500 BP in Tunisia [62], and ~7400−7000 BP in Algeria [63] and Morocco [59,64,65]. The arrival and dispersal routes of these Neolithic communities have been widely debated [62,66], with proposed scenarios ranging from a dual-dispersal model to either an Iberian [59,67] or an African diffusion [57]. Our data suggests that African goat introgression was not present in the Early Neolithic in the region around Cueva de los Murciélagos; Murciélagos1 does not show genetic affinity with African breeds compared to European breeds (Fig S14 in S1 File). However, we note that Murciélagos1 has low sequencing depth, and additional data may refine our understanding. The earliest evidence of African human gene-flow in Iberia, around 4,500 years BP, comes from Camino de las Yeseras in central Iberia, with a human male carrying African ancestry [68], contemporaneously ivory and ostrich eggshells appear in Iberian tombs further elucidating African influence in Iberia during this period [59]. This, together with the absence of African goat affinity in Murciélagos1 (Figs S13 and S14 in S1 File) may suggest that there was no Africa-to-Iberia migration (or movement of goats) in the Early Neolithic of Iberia, and that the population ancestral to Murciélagos1 had a European rather than African origin. Despite this absence of African ancestry in both Iberian humans and goats, Neolithic European farmer ancestry was detected across the Strait of Gibraltar around 7,400 BP [59,69], suggesting a unidirectional connection from Iberia to the Tingitan Peninsula and possibly the establishment of a founding population in this region. More genetic data from livestock remains in Iberia and northwestern Africa may further contribute to resolving the debate surrounding the Neolithic dispersal routes and the contact between Iberia and northwestern Africa.

In addition to the host organism (goat), the occurrence of human and fox-aligning reads (Table S1 in S2 File) provides genetic evidence that the Murciélagos1 leather may have been handled and disturbed post-mortem. Given the absence of directly associated skeletal remains and the presence of mammal modifications (Fig 2), the leather was likely modified post-mortem by humans. While analysis of human remains are on-going, the leather was likely part of a funerary assemblage. There are many human remains within the same context (Fig 1C); the tanned nature of the leather suggests it was probably worn by one or multiple individuals, and eventually deposited as part of a person’s funerary assemblage. The handling of the goat leather resulted in exogenous contamination of human DNA, with evidence of ancient authenticity of the recovered DNA (Figs S3–S6 in S1 File). The presence of fox (or possibly canid) aligning reads is a curiosity; while there is evidence of presence of fox around Cueva de los Murciélagos; their occurrence is low during the Early Neolithic and became more common from the end of the Neolithic till the end of the Bronze Age (~5,000–3,000 years BP) [70]. The presence of fox DNA could reflect co-mingling of the leather with the remains of foxes or other canids, or more likely by the disturbance of the funerary context by wild foxes/canids during or after the Early Neolithic. The post-mortem damage and fragmentation of fox-aligned reads in Murciélagos1 (Fig S6 in S1 File) are reduced compared to goat or human-aligned reads (Figs S2–S4 in S1 File), suggestive of a more recent origin. In addition, evidence of recent fox activity has been documented in and around Cueva de los Murciélagos, including coprolites and signs of denning; suggesting that the DNA is of a more recent historical origin.

In summary, we report the first nuclear genetic data from domestic goats in Iberia, obtained from a tanned goat leather specimen dated to the late 8th millennium BP. This represents one of the oldest genome data recovered from organic remains outside of high altitude, high latitude conditions. The remarkable preservation of the specimen includes several deliberate perforations on the edges of the leather, perhaps made to allow for wearing of the leather as a garment. While the recovered data is limited, it allows to infer a post-Early Neolithic incursion of African goat ancestry to Spanish goats. Denser genetic sampling both temporally and geographically of ancient caprine remains within Iberia will refine our understanding of this process which shaped goat breeds alive today.

## Materials and methods

An in-depth description of the materials and methods utilised are provided in the SI appendix, a brief overview is provided here. The material, a tanned goat leather specimen, was consistently handled with powder-free gloves to prevent contamination. A portable digital microscope was used to distinguish between anthropogenic perforations and damage from post-depositional processes. Palaeogenetic work was performed in a dedicated ancient DNA facility in Trinity College Dublin. Researchers minimized potential contamination of the sample using personal protective equipment. DNA was extracted from the leather specimen (Fig S16 in S1 File); Three skin subsamples (identified as S1-S3) were subjected to a diluted ethanol wash and proteinase digestion. Two extraction approaches were performed for the three subsamples (S1-S3); Tubes S1 and S3 were subjected to a phenol-chloroform extraction [71], labelled PEX. Tube S2 was subjected to a purification protocol using High Pure Viral Nucleic Acid Large Volume silica-based spin columns [72], labelled MEX. Tubes S1-2 were USER treated [73], S3 was not USER treated; this resulted in three different aliquots 1) Partial UDG treatment PEX, 2) partial UDG treatment MEX, and 3) non-UDG treatment PEX. Double-stranded DNA (dsDNA) libraries were then constructed [30]. Sequencing was performed on an Illumina NovaSeq 6000 (2 × 100 bp).

Quality control was performed with [74,75], trimming and collapsing of the reads was performed with AdapterRemoval v2.3.1 [76]. Taxonomic composition and contamination were assessed using FastQ Screen v0.15.2 [31], which identified goat, human and fox-associated reads. Collapsed reads were aligned to the goat reference genome ARS1 [77], human reference genome hg38, and fox reference genome (VulVul2−2) with BWA aln [78], using parameters tailored for ancient DNA (-l 1024 -n 0.01 -o 2) [39]. Reads aligning to either human or fox were removed from the goat ARS1 bam file (Table S1 in S2 File). Reads aligning to human and goat were removed from the fox bam file, and reads aligning to fox and goat were removed from the human bam file (Table S4 in S2 File). MapDamage [79] was applied to assess aDNA damage patterns (Figs 2 and S2 in S1 File).

A projection Principal Component Analysis (PCA) was performed using LASER v2.4 [34]. The PCA reference space and projection transformation were constructed using the VarGoats dataset [35], filtered to retain transversions with a MAF of 5%, resulting in a total of 2,420,382 SNPs, as in [80]. Transversion pseudohaploid genotypes were called using ANGSD v0.941-11-g7a5e0db [81], with the -doHaploCall function, restricting the analysis to 8,026,660 autosomal transversion SNPs from the VarGoats dataset. Genotypes were converted with haploToPlink, then merged with the VarGoats dataset, and converted to EIGENSTRAT using ADMIXTOOLS’ convertf tool [82]. These files were used to compute outgroup *f*_*3*_ statistics [83] and *D* statistics with ADMIXTOOLS version 7.0.2 [82]. Granular breed groupings were used, with domestic sheep as the outgroup. For mitochondrial DNA, collapsed reads were aligned using bwa aln to a circularized version of the goat mitochondrial genome (NC_005044.2), followed by a realignment to a circularized haplogroup representative; consensus sequences were generated with ANGSD v0.941-11-g7a5e0db. Mitochondrial sequences were aligned using MAFFT v7 [84]. Mitochondrial trees were built with RAxML v8.2.12 [85] under the GTR+Gamma substitution model.

To characterize the microbiome, host reads were removed by aligning sequences with Bowtie2 v2.4.4 [86] against a concatenated reference of human and several animal genomes. Unmapped reads were analyzed with KrakenUniq v1.0.4 [87] for metagenomic profiling using a custom database [88]. Radiocarbon dating was performed at the Tandem Laboratory, Uppsala University. Lipids were extracted, and the insoluble fraction was isolated through acid and base treatments, then dried and combusted for ¹⁴C analysis. The resulting date (Ua-78252) was calibrated in OxCal v4.4.4 using the IntCal20 curve [29,30].

### Significance

We report and analyze the exceptional archaeological specimen – a 7,250−7,000-year-old goat leather object, recovered from the Early Neolithic site of Cueva de los Murciélagos located in Albuñol, Spain, We additionally present genetic data from the specimen, representing one of the oldest low-altitude genomes obtained from preserved organic remains. Archaeozoological and radiocarbon analyses confirm its age, and anthropogenic treatment indicating its possible use as a garment or funerary object. Genetic analysis identifies the leather’s source as a domestic goat, with shared ancestry spanning 7,000 years to modern Bermeya goats from northern Spain. Together, these findings provide a rare, direct glimpse into Neolithic animal management and its enduring legacy in present-day Iberia.

## Supporting information

S1 File Supplementary methods and figs S1-16. These are the supplementary methods and SI figs S1-16.
(DOCX)

S2 File Supplementary Tables S1-10. These are the supplementary Tables S1-10.
(XLSX)
