# Supplementary material for: Genetic analysis of 7,000 year old preserved goat leather from Cueva de los Murciélagos (Albuñol, Spain)
Source: PLoS One. 2026 Nov 4;21(11):e0358468. doi: 10.1371/journal.pone.0358468 (PMC13734488; doi:10.1371/journal.pone.0358468)
Supplement: Supplementary file 2 [file pone.0358468.s004.docx]

Journal Requirements:

When submitting your revision, we need you to address these additional requirements.

1. Please ensure that your manuscript meets PLOS ONE's style requirements, including those for file naming. The PLOS ONE style templates can be found at <https://journals.plos.org/plosone/s/file?id=wjVg/PLOSOne_formatting_sample_main_body.pdf> and [https://journals.plos.org/plosone/s/file?id=ba62/PLOSOne_formatting_sample_title_authors_affiliations.pdf](https://track.editorialmanager.com/CL0/https:%2F%2Fjournals.plos.org%2Fplosone%2Fs%2Ffile%3Fid=ba62%2FPLOSOne_formatting_sample_title_authors_affiliations.pdf/1/010f019f65274499-0f3c9572-10e7-4aff-aaa9-0be372cfc52c-000000/BNuE8Lu-pU6y79Qm5p3KmWe3Bd78HdrGNGw3SoTAHMY=258)

2. Thank you for stating in your Funding Statement:

[This research has been carried out within the framework of different research projects: ‘De los museos al territorio: actualizando el estudio de la Cueva de los Murciélagos of Albuñol (Granada)’ (MUTERMUR) (CM/JIN/2021-009) and ‘La Cueva de los Murciélagos of Albuñol una ventana única a la Prehistoria del sur de Europa’ (MUTERMUR 2.0) (CM/DEMG/2024-046) financed by the Comunidad de Madrid and Universidad de Alcalá (directed by F.M.S.). Fieldwork at the site was conducted with permission from Delegación Territorial de la Junta de Andalucía de Cultura y Patrimonio Histórico de Granada (EXP: BC.03.143/22 14100 and EXP: BC.03.136/23 15490). We would like to thank all the volunteers and students involved in the fieldwork, and especially technician Sergio Fernández Martín for his supervision and willingness during the work. P.H.V is supported by a postdoctoral contract Juan de la Cierva by the Ministerio de Ciencia, Innovación y Universidades y Agencia Estatal de Investigación (AEI) (JDC2023-051629) and RMMS´s work is part of the project “First Settlements in Inland/Central Andalusia” (ERSAND) [Ref. PID2023-152309NA-I00 / funded by the Ministry of Science, Innovation and Universities of the Government of Spain]. This publication has emanated from research conducted with the financial support of Taighde Éireann – Research Ireland under Grant number 21/PATH-S/9515(T); and was supported by the European Research Council under the European Union’s Horizon 2020 research and innovation program (grant no. 885729-AncestralWeave).].

Please provide an amended statement that declares *all* the funding or sources of support (whether external or internal to your organization) received during this study, as detailed online in our guide for authors at <http://journals.plos.org/plosone/s/submit-now>. Please also include the statement “There was no additional external funding received for this study.” in your updated Funding Statement.

Please include your amended Funding Statement within your cover letter. We will change the online submission form on your behalf.

We have supplied this in the cover letter.

3. Please remove any funding-related text from the manuscript. Please note that funding information should not appear in any section or other areas of your manuscript. We will publish the funding statement that is present in the Financial Disclosure section of the online submission form.

This has been removed.

4. Please include a complete copy of PLOS’ questionnaire on inclusivity in global research in your revised manuscript. Our policy for research in this area aims to improve transparency in the reporting of research performed outside of researchers’ own country or community. The policy applies to researchers who have travelled to a different country to conduct research, research with Indigenous populations or their lands, and research on cultural artefacts. The questionnaire can also be requested at the journal’s discretion for any other submissions, even if these conditions are not met. Please find more information on the policy and a link to download a blank copy of the questionnaire here: <https://journals.plos.org/plosone/s/best-practices-in-research-reporting>. Please upload a completed version of your questionnaire as Supporting Information when you resubmit your manuscript.

We have submitted the required questionnaire with this submission.

5. In your manuscript, please provide additional information regarding the specimens used in your study. Ensure that you have reported human remain specimen numbers and complete repository information, including museum name and geographic location.

If permits were required, please ensure that you have provided details for all permits that were obtained, including the full name of the issuing authority, and add the following statement:
“All necessary permits were obtained for the described study, which complied with all relevant regulations.”

If no permits were required, please include the following statement:
“No permits were required for the described study, which complied with all relevant regulations.”

This is added in the data availability section.

For more information on PLOS One's requirements for paleontology and archeology research, see [https://journals.plos.org/plosone/s/submission-guidelines#loc-paleontology-and-archaeology-research](https://track.editorialmanager.com/CL0/https:%2F%2Fjournals.plos.org%2Fplosone%2Fs%2Fsubmission-guidelines%23loc-paleontology-and-archaeology-research/1/010f019f65274499-0f3c9572-10e7-4aff-aaa9-0be372cfc52c-000000/rWbyMicgwYuZQt9uZh2WXRc3iOpaZk9cdsmO17e7xKw=258).

6. One of the noted authors is a group or consortium [The VarGoats consortium]. In addition to naming the author group, please list the individual authors and affiliations within this group in the acknowledgments section of your manuscript. Please also indicate clearly a lead author for this group along with a contact email address.

We have added this in the acknowledgements

7. Please upload a new copy of Figure 1 as the detail is not clear. Please follow the link for more information: [https://journals.plos.org/plosone/s/figures](https://track.editorialmanager.com/CL0/https:%2F%2Fjournals.plos.org%2Fplosone%2Fs%2Ffigures/1/010f019f65274499-0f3c9572-10e7-4aff-aaa9-0be372cfc52c-000000/mcRqba3qDXQIcNSu7ewtMJX5hCMer9vsXQBw_sruM2c=258)

Done.

8. We note that Figures 1, 2, and S16 in your submission contain images which may be copyrighted. All PLOS content is published under the Creative Commons Attribution License (CC BY 4.0), which means that the manuscript, images, and Supporting Information files will be freely available online, and any third party is permitted to access, download, copy, distribute, and use these materials in any way, even commercially, with proper attribution. For more information, see our copyright guidelines: <http://journals.plos.org/plosone/s/licenses-and-copyright>.

We require you to either (1) present written permission from the copyright holder to publish these figures specifically under the CC BY 4.0 license, or (2) remove the figures from your submission:

1. You may seek permission from the original copyright holder of Figures 1, 2, and S16 to publish the content specifically under the CC BY 4.0 license.

We recommend that you contact the original copyright holder with the Content Permission Form (<http://journals.plos.org/plosone/s/file?id=7c09/content-permission-form.pdf>) and the following text:

“I request permission for the open-access journal PLOS ONE to publish XXX under the Creative Commons Attribution License (CCAL) CC BY 4.0 (<http://creativecommons.org/licenses/by/4.0/>). Please be aware that this license allows unrestricted use and distribution, even commercially, by third parties. Please reply and provide explicit written permission to publish XXX under a CC BY license and complete the attached form.”

Please upload the completed Content Permission Form or other proof of granted permissions as an "Other" file with your submission.

In the figure caption of the copyrighted figure, please include the following text: “Reprinted from [ref] under a CC BY license, with permission from [name of publisher], original copyright [original copyright year].”

2. If you are unable to obtain permission from the original copyright holder to publish these figures under the CC BY 4.0 license or if the copyright holder’s requirements are incompatible with the CC BY 4.0 license, please either i) remove the figure or ii) supply a replacement figure that complies with the CC BY 4.0 license. Please check copyright information on all replacement figures and update the figure caption with source information. If applicable, please specify in the figure caption text when a figure is similar but not identical to the original image and is therefore for illustrative purposes only.

We have obtained the permissions for Figures 1 and 2 and added them as a PDF file (Authorization to use the original topographic map of Cueva de los Murciélagos.pdf). For Figure S16, we have added "photo by" credits, as the photos were taken as part of the study. The photos were taken by two authors of the paper, Kevin Daly and Valeria Mattiangeli.

9. We note that Figures 1 and 5 in your submission contain map images which may be copyrighted. All PLOS content is published under the Creative Commons Attribution License (CC BY 4.0), which means that the manuscript, images, and Supporting Information files will be freely available online, and any third party is permitted to access, download, copy, distribute, and use these materials in any way, even commercially, with proper attribution. For these reasons, we cannot publish previously copyrighted maps or satellite images created using proprietary data, such as Google software (Google Maps, Street View, and Earth). For more information, see our copyright guidelines: <http://journals.plos.org/plosone/s/licenses-and-copyright>.

We require you to either (1) present written permission from the copyright holder to publish these figures specifically under the CC BY 4.0 license, or (2) remove the figures from your submission:

We are using the R Natural Earth package, so all these maps are in the public domain and are not under copyright.

1. You may seek permission from the original copyright holder of Figures 1 and 5 to publish the content specifically under the CC BY 4.0 license.

We recommend that you contact the original copyright holder with the Content Permission Form (<http://journals.plos.org/plosone/s/file?id=7c09/content-permission-form.pdf>) and the following text:

“I request permission for the open-access journal PLOS ONE to publish XXX under the Creative Commons Attribution License (CCAL) CC BY 4.0 (<http://creativecommons.org/licenses/by/4.0/>). Please be aware that this license allows unrestricted use and distribution, even commercially, by third parties. Please reply and provide explicit written permission to publish XXX under a CC BY license and complete the attached form.”

Please upload the completed Content Permission Form or other proof of granted permissions as an "Other" file with your submission.

In the figure caption of the copyrighted figure, please include the following text: “Reprinted from [ref] under a CC BY license, with permission from [name of publisher], original copyright [original copyright year].”

2. If you are unable to obtain permission from the original copyright holder to publish these figures under the CC BY 4.0 license or if the copyright holder’s requirements are incompatible with the CC BY 4.0 license, please either i) remove the figure or ii) supply a replacement figure that complies with the CC BY 4.0 license. Please check copyright information on all replacement figures and update the figure caption with source information. If applicable, please specify in the figure caption text when a figure is similar but not identical to the original image and is therefore for illustrative purposes only.

The following resources for replacing copyrighted map figures may be helpful:

USGS National Map Viewer (public domain): <http://viewer.nationalmap.gov/viewer/>
The Gateway to Astronaut Photography of Earth (public domain): <http://eol.jsc.nasa.gov/sseop/clickmap/>
Maps at the CIA (public domain): <https://www.cia.gov/library/publications/the-world-factbook/index.html> and [https://www.cia.gov/library/publications/cia-maps-publications/index.html](https://track.editorialmanager.com/CL0/https:%2F%2Fwww.cia.gov%2Flibrary%2Fpublications%2Fcia-maps-publications%2Findex.html/1/010f019f65274499-0f3c9572-10e7-4aff-aaa9-0be372cfc52c-000000/RyDMl5w1sI2lTB9BaowUInmwqKTTTMxETJlDAbBv3i8=258)
NASA Earth Observatory (public domain): <http://earthobservatory.nasa.gov/>
Landsat: [http://landsat.visibleearth.nasa.gov/](http://track.editorialmanager.com/CL0/http:%2F%2Flandsat.visibleearth.nasa.gov%2F/1/010f019f65274499-0f3c9572-10e7-4aff-aaa9-0be372cfc52c-000000/tRF3dElu_XBcnQRgjQW2l6eem8miI5UXLyDqjuF4Z7c=258)
USGS EROS (Earth Resources Observatory and Science (EROS) Center) (public domain): <http://eros.usgs.gov/#>
Natural Earth (public domain): [http://www.naturalearthdata.com/](http://track.editorialmanager.com/CL0/http:%2F%2Fwww.naturalearthdata.com%2F/1/010f019f65274499-0f3c9572-10e7-4aff-aaa9-0be372cfc52c-000000/YaJkWqFf1-dYgP5MM9UzIxWgjl9ogCTmD3PbTX1liXs=258)

We are using the R Natural Earth package, so all these maps are in the public domain.

10. Please include captions for your Supporting Information files at the end of your manuscript, and update any in-text citations to match accordingly. Please see our Supporting Information guidelines for more information: [http://journals.plos.org/plosone/s/supporting-information](http://track.editorialmanager.com/CL0/http:%2F%2Fjournals.plos.org%2Fplosone%2Fs%2Fsupporting-information/1/010f019f65274499-0f3c9572-10e7-4aff-aaa9-0be372cfc52c-000000/fMc8-EBkD8ayYfw79U4spaJJ_gMbCd9ULV5q0DrOyYk=258).

Please review your reference list to ensure that it is complete and correct. If you have cited papers that have been retracted, please include the rationale for doing so in the manuscript text, or remove these references and replace them with relevant current references. Any changes to the reference list should be mentioned in the rebuttal letter that accompanies your revised manuscript. If you need to cite a retracted article, indicate the article’s retracted status in the References list and also include a citation and full reference for the retraction notice.
We have checked all references, and added captions.

Reviewers' comments:

**Comments to the Author**

1. Is the manuscript technically sound, and do the data support the conclusions?

The manuscript must describe a technically sound piece of scientific research with data that supports the conclusions. Experiments must have been conducted rigorously, with appropriate controls, replication, and sample sizes. The conclusions must be drawn appropriately based on the data presented.

Reviewer #1: Yes

Reviewer #2: Yes

2. Has the statistical analysis been performed appropriately and rigorously?

Reviewer #1: I Don't Know

Reviewer #2: Yes

3. Have the authors made all data underlying the findings in their manuscript fully available?

The [PLOS Data policy](http://track.editorialmanager.com/CL0/http:%2F%2Fwww.plosone.org%2Fstatic%2Fpolicies.action%23sharing/1/010f019f65274499-0f3c9572-10e7-4aff-aaa9-0be372cfc52c-000000/624JJJeVc8NTS_lgVtA1S_ZsOJ7wd90ZGqv4jV5IDrI=258) requires authors to make all data underlying the findings described in their manuscript fully available without restriction, with rare exception (please refer to the Data Availability Statement in the manuscript PDF file). The data should be provided as part of the manuscript or its supporting information, or deposited to a public repository. For example, in addition to summary statistics, the data points behind means, medians and variance measures should be available. If there are restrictions on publicly sharing data—e.g. participant privacy or use of data from a third party—those must be specified.

Reviewer #1: Yes

Reviewer #2: Yes

4. Is the manuscript presented in an intelligible fashion and written in standard English?

PLOS ONE does not copyedit accepted manuscripts, so the language in submitted articles must be clear, correct, and unambiguous. Any typographical or grammatical errors should be corrected at revision, so please note any specific errors here.

Reviewer #1: Yes

Reviewer #2: Yes

5. Review Comments to the Author

Please use the space provided to explain your answers to the questions above. You may also include additional comments for the author, including concerns about dual publication, research ethics, or publication ethics. (Please upload your review as an attachment if it exceeds 20,000 characters)

**Reviewer #1:** The article entitled” Genetic analysis of 7,000 year old preserved goat leather from Cueva de los Murciélagos (Albuñol, Spain)” makes a significant contribution to our understanding of livestock management in early Neolithic Iberia.
It is focused in a detailed DNA analysis indicating that the skin fragments belong to domestic goats (Capra hircus). Furthermore, they link them primarily to European goats, particularly to the Bermeya breed from northern Iberia. The discussion arising from this link is an important contribution to the debate on how the Neolithic expansion unfolded in the Western Mediterranean, and notably, the chronology of the relationships between southern Iberia and the northern Maghreb.

Although I am not an expert in DNA studies, I believe that the methodology employed, the databases selected and the statistical analysis are appropriate for the purposes of the article.

However, this article also provides an opportunity to address issues relating to hide/leather technology in the early Neolithic period.
I therefore believe that the authors should be more precise regarding the methodology used for the macroscopic study of the hide.

Firstly, they should be more explicit about the criteria used to identify the species. For example, a reference is made to the distribution of the follicles, but no explanation or bibliography is given as to what that distribution looks like.

We thank the reviewer for this helpful comment. To clarify the criteria used for species identification, we have expanded the corresponding section of the manuscript to describe the diagnostic hair follicle pattern observed on the grain surface of the leather and added the appropriate bibliographic reference. The revised text now reads:

"Species identification was based on the morphology and distribution of hair follicles observed on the grain surface of the leather. Goat skin is characterized by a single row of primary hair follicles associated with one or two secondary follicles, a pattern that distinguishes it from cattle and deer (Nagaraju et al. 2012)."

Nagaraju GN, Prasad RV, Jamuna KV, Ramkrishna V. Histomorphological features in the differentiation of skin of spotted deer (Axis axis), cattle (Bos indicus) and goat (Capra hircus). Indian J Vet Anat. 2012;24(1):10–12.

The description of the evidence relating to the treatment of the hide should also be more detailed. It is stated that ‘the opposite leather side displays traces consistent with tanning’.
What are these traces?

We thank the reviewer for these valuable comments. We agree that the previous wording could be interpreted as implying a level of technological characterization beyond the scope of the present study. The primary objective of this manuscript is to present the genetic analysis of the leather, whereas a comprehensive technological investigation of the hide, including its manufacture, processing, and conservation, is currently underway and will be presented in a dedicated publication.

To clarify this point, we have included the following sentence in the revised manuscript:

“The flesh side exhibits a smooth and compact surface with the absence of adhering soft tissues and a homogeneous appearance of the collagen matrix. These characteristics are consistent with processed leather (Fig.2E).”

Is this a genuine tanning? Has the addition of tanning agents or tanning preparations been detected?

We thank the reviewer for this comment. At present, we cannot identify the specific tanning technique, nor have tanning agents or tanning preparations been chemically detected. Likewise, the identification of possible mechanical treatment marks requires further microscopic and experimental analyses that are part of the ongoing study.

Have marks of mechanical treatment, beyond the perforations, been identified?

We thank the reviewer for this query. No additional marks of mechanical treatment beyond the perforations have been identified as part of the analyses presented in this study. A detailed technological investigation of the leather, including the assessment of possible mechanical treatment marks and hide-processing techniques, is currently underway.

Has any experimentation been carried out to distinguish between intentional perforations and those created by insects, or they are based in bibliographical references, and if so, which ones?

We thank the reviewer for this valuable comment. Experimental work to distinguish intentional perforations from those produced by insect activity is currently underway as part of an ongoing technological study of the leather. Therefore, the results are not yet available and cannot be included in the present manuscript. At this stage, our interpretation is based on the morphology of the perforations and comparison with published descriptions of insect damage on historical leather.

To clarify this point, we have included references describing characteristic insect-related damage, including insect holes, tunnels, fibre erosion, and other surface alterations (Nafstad et al. 2001; Mahmoud et al. 2025).

Mahmoud M, Al-Gaoudi H, Hassan R. (2025). *An analytical study of historical leather binding for manuscript "Explanation and Interpretation of Al-Safari" back to 1499 A.D., the Library of Al-Azhar Al-Sharif Mosque*. Egyptian Journal of Archaeological and Restoration Studies 15(2): 209–216.

Nafstad, O.; Wisløff, H., and Grønstøl, H. 2001. Morphology of the Leather Defect Light Flecks and Spots. Acta Veterinaria Scandinavica, 42, 107-112.

**Reviewer #2:** Genetic analysis of 7,000 year old preserved goat leather from Cueva de los Murciélagos (Albuñol, Spain)
First of all, I would like to congratulate the authors on this exceptional and meticulously executed piece of work. Although my main area of expertise lies in archaeological science and material culture rather than pure genetics, I frequently collaborate with palaeogeneticists and deeply appreciate the rigorous interdisciplinary effort required to obtain a dataset of this nature. For this reason, I do not feel sufficiently qualified to evaluate the specific methodology of the genomic analysis in depth; instead, I have focused my review on the formal, archaeological, and data-presentation aspects of the manuscript.
The successful recovery and sequencing of genome-wide data from an organic skin specimen roughly 7,000 years old is a truly extraordinary achievement. As the authors rightly point out, DNA preservation at archaeological sites in southern Iberia is exceedingly difficult due to the region's climatic conditions; this finding is therefore of great importance for obtaining genetic data within this geographic context. This study magnificently demonstrates the extraordinary preservation potential of certain Mediterranean subterranean microenvironments and how soft tissue can be exceptionally preserved outside of permafrost or high-altitude contexts.

We thank the reviewer for their kind words and positive feedback.

The historical implications of this manuscript are profound: demonstrating the early establishment of European domestic goat lineages in the Iberian Peninsula and suggesting that African genetic introgression was a post-Neolithic event provides fundamental clarity to the study of ancient animal husbandry. This work undoubtedly represents a milestone both for research into livestock dispersal from the Near East and for the study of prehistoric leather technology. It is also worth noting that the figures and tables presented are highly illustrative, offering a clear and high-quality visual representation that greatly facilitates understanding of the data presented. I would also like to explicitly highlight the rigour with which the authors have confirmed that the recovered DNA is genuinely ancient rather than modern contamination; this is a nice demonstration of good practice in this type of study.

The comparative analysis with modern breeds strikes me as particularly interesting, and I consider it one of the manuscript's strengths. This approach not only helps us better understand the historical evolution of present-day Iberian goats, but also makes a valuable contribution to the ongoing scientific debate on genetic contacts and exchanges between North Africa and the Iberian Peninsula in prehistory. As a constructive observation, I feel the number of modern breeds analysed in the comparative study is somewhat limited. On a first reading, the manuscript gives the impression that the Murciélagos1 specimen only shares affinity with the Bermeya breed. In reality, however, there is a significant number of native Iberian breeds that have not been included or tested in the statistical analyses of the work. I would recommend that the authors clearly specify the exact number of Iberian Peninsula breeds that were studied.

We thank the reviewer for his kind words. We completely agree that the number of Iberian goat breeds is limited, we would love to have more access to genome data from other native Iberian breeds, but we are limited to the breeds within the VarGoat dataset. We agree, that it is not clear how many Iberian breeds are included and that this could influence the statement. We have added text to clarify this on line 388-394

“A limitation of this study is the low representation of modern Spanish breeds, with only four breeds (two from the Iberian Peninsula, one from the Balearic Islands, and one from the Canary Islands). Future analyses with access to a broader range of native Iberian breeds may improve our understanding of the genetic affinities between Neolithic goats and modern Iberian breeds,and possibly identify regional patterns of continuity that are not captured by the current dataset.”

Along these same lines, it should be noted that the aligned reads recovered from Murciélagos1 show a short average length, and the authors are candid in acknowledging this limitation at several points throughout the discussion. That said, given that a substantial part of the manuscript's central conclusions (the affinity with Bermeya, the absence of African introgression in the Neolithic) rest on this single, highly fragmented genome, I would suggest the authors consider whether it might be worthwhile to consolidate this discussion into a single, explicit paragraph on methodological limitations, rather than mentioning it in a dispersed manner throughout the text. This would help the reader better gauge the true weight of results. It is worth emphasising, however, that although genetic results from a single specimen are not sufficient on their own to support general population-level interpretations, they are nonetheless enormously valuable as a starting point and for establishing initial trends, which one would hope future genetic studies with larger numbers of specimens will go on to confirm or refine.

We thank the reviewer for this comment and agree that explicitly discussing the methodological limitations provides important context for interpreting our findings. We have therefore added a dedicated paragraph to the Discussion (lines 324–335)

Far from being an unaddressable shortcoming, this limited representation of Iberian breeds makes this study a very interesting reference point and starting point. It would be of great interest to the scientific community to expand this work in the future, comparing the palaeogenomic data against a much broader and more complete spectrum of modern Iberian breeds, as well as against future ancient goat genomes from other Iberian sites.
Finally, there are several formal inconsistencies and typographical errors that the authors should address before publication:

We thank the reviewer for highlighting these inconsistencies and typographical errors

The Editorial Manager cover sheet does not include José Antonio Lozano Rodríguez, whereas he does appear in the manuscript (line 8, affiliation 9); both documents should be made consistent.

We thank the reviewer for notifying us. Thank you very much for flagging this to us, we will amend this with the editor.

In line 450 of the Methods, "vulvul2-2" should probably be written as "VulVul2.2", following standard genome assembly naming conventions. This has been cross-checked against the Supplementary Methods and Figures S1-16, where both spellings ("vulvul2-2" and "VulVul2.2") are in fact used in different sections, confirming this is a recurring inconsistency rather than an isolated typo.

We have amended this in the main manuscript and the SI.

Line 342 contains "Capra pyrenica" instead of "Capra pyrenaica", the correct form used elsewhere in the text (lines 118, 181, 222).

We have amended this.

Line 241 has a spacing issue ("anEast Iranian genome" should read "an East Iranian genome").

We have amended this.

I noticed that the main text refers to alignment against "hg39" (line 450), whereas the Supplementary "Data handling" section refers to "hg38". As I am not an expert in the specific bioinformatic pipeline, I was simply surprised by this discrepancy and would appreciate it if the authors could clarify whether this is a typographical error or reflects two different reference genomes used at different stages.

Thank you for pointing this out. In this specific case, it was indeed a typo, and the reference genome stated on line 450 should have been hg38 rather than hg39. However, for complete transparency, we would like to clarify that two different human reference genomes were used during this study, reflecting the fact that the data were processed by two laboratories. The Daly laboratory processed the ancient animal reads and used hg38 for the removal of potential human contamination from the samples. The Fernández-Domínguez laboratory processed the ancient human reads and used hg19 as the reference genome for their analyses.

We do not expect this difference to affect the results, as the two datasets were not directly compared with each other, and relate to different analyses.

Reference 25 (Morell Miranda et al., lines 601-604) has the journal name duplicated ("bioRxiv. bioRxiv; 2026"), likely due to a formatting error when exporting the reference list.
Reference 42 (Altamirano et al.) is missing the year, volume, and page numbers, unlike the rest of the citations in the list.

We have changed the double bioRxiv and added the complete reference to Altamirano et al.

“Altamirano M, Herrero-Otal M, Piqué R, Palomo Pérez A, Homs A, Maicas R, et al. Approaches to the osseous industry from Cueva de los Murciélagos in Albuñol (Granada, Spain) and its possible relationship with plant-based technologies. In: Goutas N, Christensen M, editors. *15e rencontre internationale du Worked Bone Research Group de l'ICAZ (Paris, 2024).* Séances de la Société préhistorique française. Paris: Société préhistorique française; 2026. p. 323–334.”

Also confirmed against the Supplementary Methods and Figures S1-16, the legends of Figures S12, S13, and S14 each contain the same wording: "the later two living roughly a millennia within Murciélagos1 estimated age." This should read "the latter two" (not "later") and "a millennium" (not "millennia," which is already plural); since the error is identical across all three legends, a single correction should resolve it in each case.
I also noticed that the legend of Figure S11 cites reference "(54)", but the Supplementary reference list only extends to reference 32. I would recommend the authors review this.

We have amended this in the SI.

I would like to reiterate my congratulations to the authors for this important contribution to our understanding of domestication and livestock management in Iberian prehistory. This is an exceptional piece of work, both for the rarity of the find and the rigour of its execution, and I believe it should be published once these minor issues have been addressed.

We again thank the reviewer for their kind words, and thank both reviewers for their time constructively reviewing the manuscript.
